# Uneven burden of multidimensional poverty in India: A caste based analysis

**DOI:** 10.1371/journal.pone.0271806

**Published:** 2022-07-29

**Authors:** Itishree Pradhan, Binayak Kandapan, Jalandhar Pradhan

**Affiliations:** Department of Humanities and Social Sciences, National Institute of Technology, Rourkela, India; Dr Baba Saheb Ambedkar Medical College and Hospital, INDIA

## Abstract

Poverty is multifaceted. The global poverty profile shows 41% of multidimensionally poor people living in South Asian countries. Though castes and tribes are a more prevalent line of social stratification in India, and their socio-economic characteristics also vary remarkably, hardly any study has explored these dimensions while analysing multidimensional poverty in India. Hence, this study attempts to assess the multidimensional status of poverty among the social groups in India. National Family Health Survey, 2015–16 (NFHS-4) is a source of rich information on 579,698 households’ well-being for this analysis. Alkire- Foster technique was applied to decompose the Multidimensional Poverty Index (M_0_) across its dimensions and indicators for all the social groups. Three broad dimensions of deprivation–Health, Education and Standard of Living–include 12 indicators, guided by the poverty literature, data availability and the country’s sustainable Development Goals (SDGs). There were three main findings in this study: (1) Scheduled Tribes (STs) are the most disadvantaged subgroup in India with remarkably high values of headcount (H = 0.444;), intensity (A = 0.486), and M_0_ (0.216), followed by Scheduled Castes (SCs) (H = 0.292; A = 0.473; M_0_ = 0.138), and Other Backward Classes (OBCs) (H = 0.245; A = 0.465; M_0_ = 0.114); and Others category is the most privileged with very low values of H = 0.149, A = 0.463, and M_0_ = 0.069; (2) STs contribute nearly twice their population share for both H and M_0_, and the SCs contribution is also noticeably higher than their population share; (3) States located in the central and eastern regions of India have the higher H, A and M_0_ for all the social groups. This suggests that there is a need for a thorough assessment of poverty at specific levels to uncover the poverty situation in society, improve the effectiveness of evidence-based planning and effective policymaking.

## 1. Introduction

Traditionally ‘Poverty’ is defined as scarcity of income by most of the countries worldwide [1] and it is understood by the financial status of a person or a household or a community [2]. Sen [3] described the income or unidimensional poverty measurement in his monumental work “Poverty: An Ordinal Approach to Measurement,”. However, he acknowledged that it is difficult to replicate this ordinal approach in day to day life. Since the mid-1970s, it has been widely accepted that poverty is nothing but the low level of income [4]. However, studies have shown that income cannot be used to determine a person’s well-being [2], because it does not account for nonmonetary deprivations such as lack of access to nutritious food, healthcare services, better education, safe drinking water, improved sanitation facilities, clean cooking fuel, electricity, adequate housing condition, financial security, basic information and so on, and thus fails to identify who is poor [3,5–8]. With time, various ways for identifying the poor gained prominence, including the ‘basic needs approach’, ‘social exclusion’, and ‘capability approach’. As a result, researchers have combined monetary and nonmonetary indices of deprivation to produce a more comprehensive picture of poverty [7]. In the word of Sen [9], “Human lives are battered and diminished in all kinds of different ways, and the first step is to accept that deprivations of very different kinds have to be accommodated within a general overarching framework”. Hence it is suggested that the multidimensional poverty measurement is more reliable than unidimensional poverty measurement [10–12]. Consequently, the poverty argument has shifted from a unidimensional (income) to a multidimensional prospective, resulting in a more comprehensive picture of poverty [1]. The Sustainable Development Goals (SDGs) of the United Nations (UN), often known as the 2030 Agenda for Sustainable Development, have reignited interest in multidimensional poverty reduction strategies [13]. The SDGs target 1.2 says “by 2030, reduce at least half the proportion of men, women and children of all ages living in poverty in all its dimensions according to national definitions” [14].

The global multidimensional poverty index in 2021 covers 5.9 billion people of 109 countries worldwide: 26 low-income, 80 middle-income and three high-income. The findings indicate one in every five persons are multidimensionally poor in these 109 countries combined, which represents approximately 1.3 billion people, and 41% (532 million) of them are living in the South Asian countries [15]. Despite experiencing the largest reduction in the number of multidimensionally poor people during the decade 2006–16 [16], India has more than 381 million of the multidimensionally poor, accounting for a whopping proportion of 72% multidimensionally poor in South Asia [15]. Though, India’s major economic growth over the past 30 years has continued to lift millions of people out of poverty, the unforeseen impact of COVID-19 may have potentially pushed more people into poverty [15]. Because, many people lost their jobs; they were unable to meet their essential needs; their consumption expenditure reduced; and they became impoverished. As a result, India may witness a significant increment in its poverty rate in the near future.

The household has been utilised as a unit of analysis in the majority of empirical studies on the multidimensional poverty measure [17–19]. Which considers all members of the household as multidimensionally poor if the household is multidimensionally poor [18,20]. The MPI creates a vivid image of household living in poverty within and across countries, regions and the world [21]. It calculates the severity of deprivation in poor populations using various deprivation indices [22–24]. In India, numerous studies have already been conducted using the multidimensional approach to define poverty and its trends and determinants. These studies are basically estimated poverty across the states and regions in India [25–32].

In India, the Scheduled Castes (SCs), and Scheduled Tribes (STs) minorities have historically been the most underprivileged [33]. Because, these groups have faced discrimination, and thus exclusion, in one way or another from the mainstream economic and social spheres. Despite the fact that the government initiated various projects and programmes for the uplift of these groups, which were backed by substantial financial support, their growth in terms of economic and social status has still stagnant [34]. According to global MPI estimates, five out of every six multidimensionally poor in India belongs to SC, ST or Other Backward Class (OBC) households: ST with more than 50% multidimensionally poor, followed by SC with 33.3% and OBC with 27.2% [15] Though castes and tribes are a more prevalent line of social stratification in India, and their socio-economic characteristics also varies remarkably, there is hardly any study that explored these dimensions while analyzing multidimensional poverty in India. Hence, this study attempts to assess the differential in multidimensional poverty amongst the social groups in India. Further, it also attempts to decompose the MPI across its dimensions and indicators for all the social groups.

## 2. Deprivation, dimensions and indicators

The UN’s proposal for a globally agreed upon definition of poverty justifies the selection of the dimensions and indicators for this study. According to UN’s proposal, all basic human rights and needs, such as access to nutritious food, safe drinking water, appropriate sanitation, proper health care facilities, shelter, and education, should be considered in the poverty analysis [14]. Further, the SDGs are also useful for determining which aspects and indicators should be utilised to measure poverty in a multidimensional prospective. Access to safe drinking water, appropriate sanitation, and primary education are all goals that must be met under SDGs. Based on these human’s need and necessities MPI was calculated using data from 12 indicators across three key dimensions: health, education and standard of living, all of which were given equal weightage. Selection of the 12 indicators for the three dimensions was adopted from National Institution for Transforming India (NITI) Aayog’s report [35]. The rationale behind selection of the 12 indicators is explained in details elsewhere [35]. Detailed information on the thresholds for poverty dimensions and indicator weights are shown in Table 1.

**Table 1:** Dimensions, indicators, deprivation cut-offs, and indicators’ weight.

Dimension (Weightage)	Indicator	Deprivation cut off (a household is deprived if….)	Weight
**Health (1/3)**	Nutrition	Any child (0–59 months) or woman (15–49 years) or man (15–54 years) is found to be undernourished	1/6
	Child & Adolescent mortality	A usual resident under 18 years of age has died in the family in the 5-year period of preceding the survey.	1/12
	Maternal Health	Any woman in the household who has given birth in the five years preceding the survey and has not had at least four antenatal care visits or assistance from trained professional medical staff during the most recent childbirth.	1/12
**Education (1/3)**	Years of Schooling	None of the household member aged 10 years or older has completed 6 years of schooling.	1/6
	school attendance	Any school aged child is not attending school up to the age at which he/she would complete class 8.	1/6
**Standard of Living (1/3)**	Cooking fuel	Cooks with only unclean fuel i.e., dung, agricultural crops, shrubs, wood, charcoal or coal.	1/21
	Sanitation	The household has unimproved or no sanitation facility or it is improved but shared with other households.	1/21
	Drinking water	It does not have access to safe drinking water, which takes at most 30-minutes of walk from home.	1/21
	Electricity	It has no electricity.	1/21
	Housing	It has inadequate housing condition: floor/roof/wall is made on natural/rudimentary materials.	1/21
	Assets	Does not own a car or truck and does not own more than one of these assets: radio, TV, telephone, computer, animal cart, bicycle, motor bike, and refrigerator	1/21
	Bank account	No household member has a bank account.	1/21

**Source:** Adapted from NITI Aayog [35].

### 2.1. Health

Health is considered as the central capability of overall well-being, and being healthy is not only a valuable achievement in itself, but also can help individuals to do many important things such as participating in social and sports activities [9]. Not being in good health often affects other capabilities such as hindering in the educational achievement and economic participation. Following the NITI Aayog report [35], the study includes nutrition, child & adolescent mortality and maternal health in the health domain. While, nutrition and child & adolescent mortality parameters are based on global MPI, the maternal health indicator is unique to India’s MPI. The maternal health indicator is a signatory to the Goal 3 of SDGs “Ensure healthy lives and promote well-being for all at all ages”, that intends to ensure rigorous adherence to the SDG targets of lowering maternal mortality and eliminating preventable new-born deaths [35]. The maternal health indicator is a union of two distinct components: Antenatal care (ANC) and assisted delivery, both of which are critical prerequisites for mothers and newborns to have a positive health outcome. With a significant percentage of maternal deaths occurring during the period of pregnancy, the four-visit antenatal care model outlined in the World Health Organization (WHO) clinical guidelines has been instrumental in the early identification of complications in pregnancy, monitoring of foetal growth and the management of complications through the referral of mothers to the appropriate facility for further treatment. The complications due to prematurity, intrapartum deaths, and neonatal infections accounts for nearly 80 percent of the new-born deaths, that can be identified and addressed for preventing death or life-long disability [36]. Since, ANC cannot be considered as prevention of intrapartum deaths, which requires quality care provided during childbirth, that is mostly characterized as the assistance of skilled health personnel during childbirth [35].

The indicators in the Health dimension are not evenly weighted. Malnutrition has significant consequences to early childhood development as well as to the health and overall wellbeing of adults, and nutritional status of an individual can be linked to almost all socio-economic development indicators. Hence, giving nutrition indicator a higher weight is justifiable. Nutrition carries half the dimension weight of 1/3 with a weight of 1/6. Similarly remaining dimension weight is divided evenly between Child-Adolescent Mortality and Maternal Health, with each indicator receiving a 1/12 weight.

### 2.2. Education

Like health, education is an important capability for improving people’s wellbeing. In most countries, especially low-income developing countries, people with more education earn more than those with less education [17]. At the very least, education has a private benefit, such as enabling people to take an active role in their social, economic, and political lives [37]. The two indicators selected under this dimension are in line with the goal 4 of SDGs “Ensure inclusive and equitable quality education and promote lifelong learning opportunities for all”, which is represented by school attendance of 6–14 years children and years of schooling of 10 years and above household members, with each indicator weighted at 1/6, representing half of the dimension weight (1/3). It is worth noting that, due to the nature of the indicator, an individual living in a ‘household with at least one member with six years of schooling’ is considered non-deprived, even if they haven’t attended school themselves. Because, even if one member of a family has more than six years of schooling, the positive effect of that education, be in terms of an increase in economic opportunities such as the ability to enter high-paying employment or improve social standing, is shared by all members of the household [35]. Similarly, an individual living in a ‘household where at least one child does not attend school’ is declared deprived in this indicator, even if they have completed their schooling. The reason for this is that a child who does not attend school is indicative of a larger set of deprivations that the family is experiencing, which acts as a barrier to the child’s education.

### 2.3. Standard of living

The ‘Standard of Living’ dimension includes several indicators that depict households’ living conditions. In this study, the ‘Standard of Living’ dimension includes seven metrics that describe a household’s access to essential services like electricity, clean cooking fuel, safe drinking water, improved sanitation, pucca housing (good flooring, roof and walls), bank account, and household assets. Except for the indicator for bank accounts, which is unique to India’s national MPI [35], all other indicators follow global definitions and cut-offs. A household’s access to a bank account is critical for availing the benefits of several flagship government programs aimed at reducing poverty, increasing access to education, and creating livelihoods–which often utilize direct benefit transfers.

It is well laid down that access to basic amenities such as safe drinking water, improved sanitation facilities, shelter, electricity, and improved cooking fuel are vital for the overall growth and development of any individual and eventually boost the economic growth of the respective country/ state [38]. The safe drinking water and improved sanitation facility are in line with the goal 6 of SDGs, that is, ensuring “availability and sustainable management of water and sanitation for all” [14]. Similarly, cooking fuel and electricity falls under the broad umbrella of goal 7 “Ensure access to affordable, reliable, sustainable and modern energy for all”, housing under the goal 11 “Make cities and human settlements inclusive, safe, resilient and sustainable”, assets and banking under the goal 1 “End poverty in all its forms everywhere” [14]. The dimension weight (1/3) is distributed evenly among the seven indicators, giving each a weight of 1/21.

## 3. Data and methodology

### 3.1. Data

**Fig 1:**
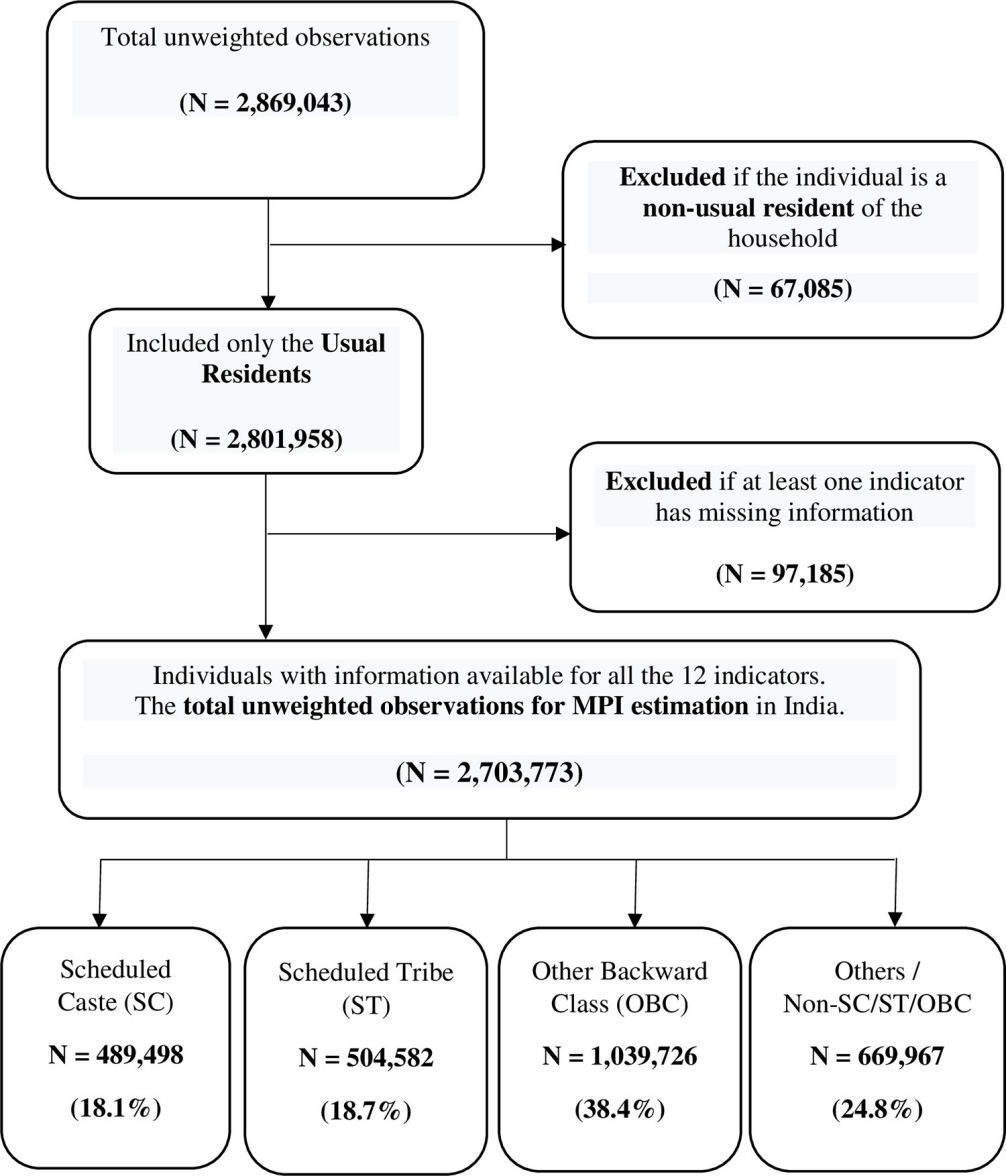
Sample selection flow chart.

This study employs micro survey data from a nationally representative Indian Demographic Health Survey collected in 2015–16. The Indian DHS is the National Family Health Survey (NFHS), which is conducted by the International Institute of Population Sciences (IIPS) under the Ministry of Health and Family Welfare (MoHFW), Government of India [39]. The NFHS-4 survey covered a nationally representative sample of 2,869,043 individuals across 628,892 households. This study has collected precise data on health, nutritional status, mortality, sociodemographic characteristics, access to basic facilities, and household assets, all of which are required for MPI calculations. In this survey, the many caste groups are grouped into four major categories: SC, ST, OBC, and Others [39]. While SC includes Dalit communities, the category ‘Other’ include higher castes. The SC and ST communities have tended to be the most disadvantaged subgroups in India, followed by the OBC community. This study is based on a total of 2,703,773 individuals from 579,698 households. Detailed procedure of sample selection for this study is depicted in Fig 1.

### 3.1. Methodology

In 2010 the Oxford Poverty and Human Development Initiative (OPHI) and United Nations Development Program (UNDP) have launched the ‘global Multidimensional Poverty Index (MPI)’. It assesses the intricacies of poor people’s lives, both individually and collectively, each year and focuses on how multidimensional poverty has decreased. It represents a deprivation in the basic rights and needs of the people; it reveals a pattern of poverty other than income poverty [22] and its indicators are selected based on theSDGs.

While using the multidimensional poverty index technique, it is important to consider the magnitude of the dimensions and indicators of poverty. Because all three dimensions are equally vital, they are given the same weight. Education, standard of living, and health are the three key deprivation indicators analysed using the AF technique [21] These dimensions were also included in this study. After that, the indicators’ weights under each dimension are assigned. Since there are different numbers of indicators under each dimension, the indicators’ weight was unevenly assigned. As a result, the index for each individual ‘i’ is a number between 0 and 1.


$${Deprivation\;Score}_{i}=\frac{1}{3}{Health}_{i}+\frac{1}{3}{Education}_{i}+\frac{1}{3}{Standard\;of\;Living}_{i}$$ (1)

Where,

$$\frac{1}{3}{Health}_{i}=\frac{1}{6}{nutrition}_{i}+\frac{1}{12}{child-adolescent\;mortality}_{i}+\frac{1}{12}{maternal\;health}_{i}$$

$$\frac{1}{3}{Education}_{i}=\frac{1}{6}{years\;of\;schooling}_{i}+\frac{1}{6}{school\;attendance}_{i}$$

$${\frac{1}{3}Standard\;of\;Living}_{i}=\frac{1}{21}{cooking\;fuel}_{i}+\frac{1}{21}{sanitation}_{i}+\frac{1}{21}{drinking\;water}_{i}+\frac{1}{21}{electricity}_{i}+\frac{1}{21}{assets}_{i}+\frac{1}{21}{housing}_{i}+\frac{1}{21}{bank\;account}_{i}$$

In this study, we have employed Alkire-Foster counting methodology [23] to determine the multidimensional poverty among social groups across the nation. The AF technique’s proposed conceptual framework is based on Amartya Sen’s ‘capability approach,’ which reflects capability poverty [23]. Based on this methodology MPIs are constructed using two analytical steps: identification and aggregation. In a dual cut-off identification technique, deprivation and poverty cut-offs are both employed to identify poor individuals. A deprivation cut-off is employed for each indicator to determine whether a person is deprived in that particular indicator, and each person’s final deprivation score is obtained by summing up their weighted deprivation score of all the indicators. Next, a poverty cut-off is utilised to evaluate whether a person falls into the multidimensionally poor category. If a person’s deprivation score is more than or equal to that poverty cut-off, he or she is considered multidimensionally poor. Here the poverty cut-off is denoted as ‘k’ and following the global MPI cut-off of ‘1/3’, cut-off point for being multidimensionally poor is set at k = 0.333.

Suppose the ‘q’ be the total number of individuals whose overall deprivation score is ≥ k = 0.333, and the total sample size of the study is ‘n’, the traditional headcount ratio or incidence (H) is computed as in Eq (2):

$$H=\frac{q}{n}$$ (2)

However, this traditional method cannot accurately reflect how severely each individual is affected by multidimensional poverty, and even if poor people become more deprived, for example, due to other deprivation aspects, this number will not grow [40]. AF method addressed this issue and improved this measurement by defining intensity of multidimensional poverty (A) indicates the average deprivation score of the multidimensionally poor and is expressed as follows:

$$A=\frac{c(k)}{q}=\frac{\sum_{i}^{q}C_{i}(k)}{q}$$ (3)

Where, ‘*C*_*i*_(*k*)’ is the deprivation score of the multidimensionally poor individual ‘*i*’; and ‘*q*’ ‘q’ is the number of people who were identified as multidimensionally poor.

Consequently, the AF method proposes the MPI, or the adjusted headcount ratio, *M*_0_, by multiplying the H with the A, represented in the equation below:

$$MPI=M_{0}=H\times A$$ (4)

Besides, M_0_ satisfies the axioms of population subgroup decomposability, dimensional breakdown and ordinality. Subgroup decomposability relates subgroup to overall poverty levels; dimensional breakdown relates multidimensional poverty levels to dimensional components; and ordinality allows meaningful evaluations of poverty when variables are ordinal. In this study, M_0_ was decomposed according to social groups (castes) and dimensions to facilitate comparisons of those castes and dimensions.

## 4. Results

### 4.1. Deprivation by indicators across social groups

Out of the total sample of 28,69,043, this study utilised the information of 27,03,773 for MPI estimation at national, state and social group levels. Table 2 depicts the percentage of people deprived in each indicator across the social groups and not necessarily being multidimensionally poor. It is important because it helps to target sector-specific poverty. It is observed that overall, the rate of deprivation is highest in cooking fuel, followed by sanitation, housing, and nutrition, and a similar pattern was observed across all the social groups. However, there exists a significant social group differential in terms of magnitude of deprivation by indicators. STs have dramatically higher rate of deprivation in all the indicators compared to other social groups, followed by SCs, and SCs followed closely by the OBCs. It is noteworthy that, relatively the STs lags much behind in both the indicators of education dimension and safe drinking water, assets, housing and banking indicators of standard of living dimension. Though SCs and OBCs show a similar pattern of deprivation in most indicators, the relative differences in household assets, banking, and years of schooling are notably higher among the SCs. Interestingly, although the relative difference in social groups is noticeable in the dimension of health, it is not as wide as the relative differences in the indicators of other two dimensions.

**Table 2:** Percentage of people deprived in each indicator of the MPI by social groups in India.

Dimension	Indicators	SC	ST	OBC	Others	Total
Health	Nutrition	44.2	50.4	41.6	33.4	40.8
	Child & Adolescent mortality	3.1	3.0	2.9	2.2	2.8
	Maternal Health	21.7	25.6	21.5	15.8	20.4
**Education**	Years of Schooling	16.3	23.0	13.1	8.8	13.5
	School Attendance	7.4	11.0	6.3	4.5	6.5
**Standard of living**	Cooking Fuel	67.1	83.2	57.6	44.0	58.3
	Sanitation	62.3	74.3	51.8	36.2	51.9
	Drinking Water	14.1	28.7	13.8	11.4	14.6
	Electricity	14.7	17.6	12.9	6.9	12.1
	Housing	53.1	70.7	44.1	33.2	45.5
	Assets	16.1	29.9	12.0	9.8	13.9
	Bank Account	10.8	14.1	8.7	8.8	9.7

Source: Authors’ own estimation.

### 4.2. Multidimensional poverty by social groups

#### H, A, and MPI at national level

The estimates for multidimensional poverty among the social groups of India are displayed in Table 3. It shows the headcount ratio (H), the average deprivation score of the multidimensional poor or intensity (A), the adjusted headcount ratio or the MPI (M_0_), weighted population share across the social groups in India. The headcount ratios reveal that nearly one-fourth (24.8%) of the Indian are multidimensionally poor; and among the social groups the headcount ratio estimated for the STs (44.4%) is stinkingly higher compared to the SCs (29.2%), OBCs (24.5%) and Others (14.9%). However, there exist merely any caste difference in the intensity of multidimensional poverty A, which ranges from 46.3% for Others to 48.6% for the STs. The STs exhibits the highest adjusted headcount ratio with M_0_ = 0.216, followed by SCs (0.138) and OBCs (0.114). It can be concluded from the table that STs are the most disadvantaged subgroup in India with remarkably high values of H, A, and M_0_, and the other categories are the most privileged with very low values of H, A, and M_0_. Meanwhile, the multidimensional poverty estimates for SCs and OBCs show a similar picture but are slightly favoured for OBCs.

**Table 3:** Multidimensional poverty estimates for the social groups in India.

	SC	ST	OBC	Others	Total (India)
**H**	0.292	0.444	0.245	0.149	0.248
**A**	0.473	0.486	0.465	0.463	0.471
M_0_ (MPI)	0.138	0.216	0.114	0.069	0.117
**Population share (Weighted)**	20.7%	9.4%	43%	26.9%	

**Source:** Authors’ own estimation.

#### H, A, and MPI across States and UTs

**Map 1:**
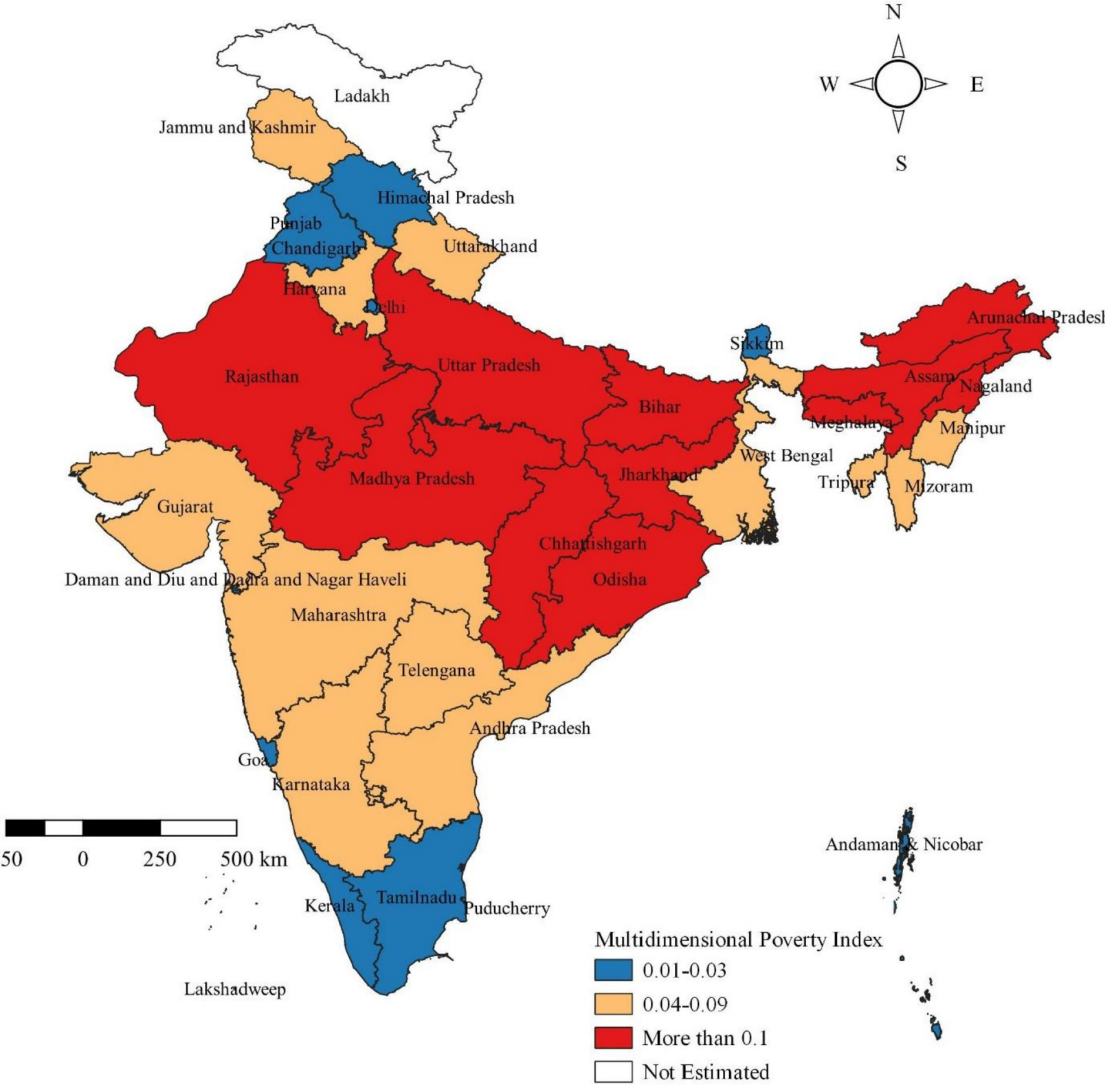
Multidimensional poverty index across the States and Union Territories in India, 2015–16. Source: Authors’ compilation based on data from NFHS-4, 2015–16. The Map was developed by the authors using QGIS Version 3.24.0, and the map was cross verified with the India map and its States and Union Territories’ boundaries as shown in the official website of Survey of India: https://indiamaps.gov.in/soiapp/.

**Map 2:**
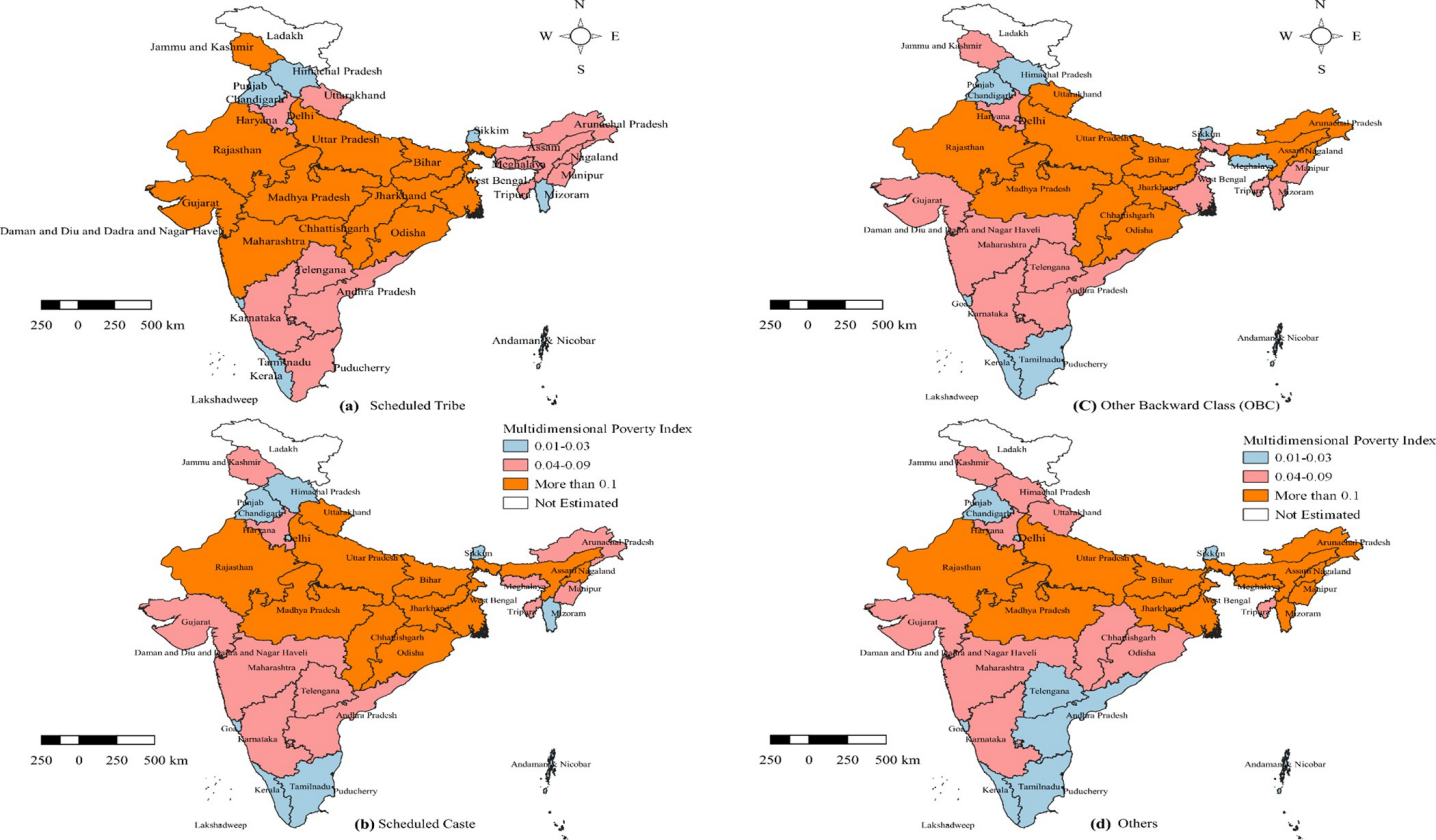
Multidimensional Poverty Index across the States and Union Territories in India by Social Groups: (a) ST (b) SC (c) OBC (d) Others, 2015–16. Source: Authors’ compilation based on data from NFHS-4, 2015–16. The Map was developed by the authors using QGIS Version 3.24.0, and the map was cross verified with the India map and its States and Union Territories’ boundaries as shown in the official website of Survey of India: https://indiamaps.gov.in/soiapp/.

Table 4 presents the H, A and MPI across the states and UTs of India by social groups. In overall, the result indicates MPI varies significantly from 0.002 in Kerala to 0.268 in Bihar. The highest MPI was estimated to be for the states of Bihar (0.268), followed by Jharkhand (0.201), Uttar Pradesh (0.18), Madhya Pradesh (0.172), Assam (0.158), Meghalaya (0.157), Rajasthan (0.139), Odisha (0.134), Chhattisgarh (0.125), Dadra & Nagar Haveli (0.121), and Nagaland (0.118). The extent of the MPI in the 11 states and UTs mentioned above was greater than at the national level. The lowest MPI was estimated to be in Kerala, followed by Puducherry, Lakshadweep, Sikkim, Goa, Delhi, and Tamil Nadu compared to other states and UTs. The states and UTs were divided into three categories according to their MPI and presented in Map 1, the poor performing states were highlighted in red, and the better-performing ones are in blue. A similar breakdown of MPI for STs, SCs, OBCs and Others were presented in Map 2(A)–2(D), respectively: orange indicates poor performers, pink indicates average performers, and blue indicates better performers.

**Table 4:** Multidimensional poverty across the States and Union Territories by social groups.

	SC			ST			OBC			Others			Total			N
	H	A	M0	H	A	M0	H	A	M0	H	A	M0	H	A	M0	
States																
**Andhra Pradesh**	0.147	0.429	0.063	0.328	0.454	0.149	0.112	0.429	0.048	0.044	0.409	0.018	0.115	0.434	0.05	36072
**Arunachal Pradesh**	0.175	0.474	0.083	0.228	0.461	0.105	0.289	0.498	0.144	0.328	0.506	0.166	0.244	0.472	0.115	56363
**Assam**	0.295	0.454	0.134	0.256	0.453	0.116	0.279	0.473	0.132	0.386	0.484	0.187	0.332	0.477	0.158	108327
**Bihar**	0.664	0.53	0.352	0.603	0.519	0.313	0.525	0.499	0.262	0.366	0.503	0.184	0.528	0.508	0.268	189124
**Chhattisgarh**	0.253	0.427	0.108	0.433	0.462	0.2	0.224	0.424	0.095	0.108	0.426	0.046	0.283	0.443	0.125	90503
**Goa**	0.03	0.367	0.011	0.083	0.398	0.033	0.016	0.375	0.006	0.036	0.389	0.014	0.035	0.396	0.014	6278
**Gujarat**	0.179	0.458	0.082	0.414	0.469	0.194	0.175	0.44	0.077	0.066	0.439	0.029	0.179	0.452	0.081	90613
**Haryana**	0.155	0.419	0.065	0.258	0.574	0.148	0.126	0.46	0.058	0.058	0.431	0.025	0.116	0.446	0.052	85466
**Himachal Pradesh**	0.113	0.407	0.046	0.104	0.394	0.041	0.048	0.396	0.019	0.056	0.393	0.022	0.071	0.395	0.028	36952
**Jammu & Kashmir**	0.187	0.444	0.083	0.346	0.471	0.163	0.096	0.417	0.04	0.088	0.432	0.038	0.122	0.441	0.054	90652
**Jharkhand**	0.507	0.495	0.251	0.56	0.496	0.278	0.371	0.453	0.168	0.226	0.46	0.104	0.423	0.476	0.201	118855
**Karnataka**	0.179	0.436	0.078	0.224	0.442	0.099	0.103	0.417	0.043	0.084	0.417	0.035	0.126	0.429	0.054	99251
**Kerala**	0.028	0.429	0.012	0.089	0.416	0.037	0.003	0.333	0.001	*- NA -*	*- NA -*	*- NA -*	0.006	0.404	0.002	44318
**Madhya Pradesh**	0.396	0.457	0.181	0.62	0.51	0.316	0.315	0.451	0.142	0.165	0.436	0.072	0.364	0.471	0.172	247115
**Maharashtra**	0.135	0.422	0.057	0.386	0.477	0.184	0.098	0.408	0.04	0.098	0.429	0.042	0.139	0.44	0.061	116627
**Manipur**	0.139	0.424	0.059	0.265	0.464	0.123	0.165	0.43	0.071	0.14	0.421	0.059	0.182	0.443	0.081	53743
**Meghalaya**	0.171	0.474	0.081	0.328	0.479	0.157	0.018	0.444	0.008	0.414	0.481	0.199	0.328	0.479	0.157	36578
**Mizoram**	0.053	0.415	0.022	0.094	0.479	0.045	0.16	0.481	0.077	0.211	0.469	0.099	0.096	0.477	0.046	50008
**Nagaland**	0.326	0.503	0.164	0.249	0.454	0.113	0.174	0.454	0.079	0.29	0.545	0.158	0.254	0.462	0.118	44319
**Odisha**	0.334	0.449	0.15	0.515	0.489	0.252	0.204	0.436	0.089	0.131	0.427	0.056	0.29	0.461	0.134	123060
**Punjab**	0.091	0.44	0.04	*- NA -*	*- NA -*	*- NA -*	0.045	0.467	0.021	0.017	0.412	0.007	0.051	0.442	0.022	75637
**Rajasthan**	0.343	0.466	0.16	0.555	0.505	0.28	0.257	0.463	0.119	0.146	0.452	0.066	0.294	0.474	0.139	167809
**Sikkim**	0.041	0.439	0.018	0.037	0.432	0.016	0.033	0.394	0.013	0.035	0.4	0.014	0.035	0.417	0.015	18561
**Tamil Nadu**	0.076	0.395	0.03	0.146	0.452	0.066	0.034	0.412	0.014	0.023	0.435	0.01	0.048	0.403	0.019	98179
**Tripura**	0.155	0.432	0.067	0.257	0.463	0.119	0.101	0.406	0.041	0.12	0.45	0.054	0.166	0.449	0.075	17299
**Uttar Pradesh**	0.475	0.478	0.227	0.632	0.516	0.326	0.396	0.47	0.186	0.224	0.473	0.106	0.38	0.474	0.18	386653
**Uttarakhand**	0.227	0.441	0.1	0.219	0.443	0.097	0.223	0.462	0.103	0.122	0.426	0.052	0.175	0.443	0.078	64299
**West Bengal**	0.232	0.448	0.104	0.415	0.465	0.193	0.159	0.434	0.069	0.186	0.457	0.085	0.21	0.453	0.095	63947
**Telangana**	0.174	0.425	0.074	0.292	0.442	0.129	0.118	0.432	0.051	0.052	0.404	0.021	0.132	0.433	0.057	27274
**Union Territories**																
**Andaman & Nicobar Islands**	0.032	0.406	0.013	0.033	0.364	0.012	0.042	0.381	0.016	0.042	0.429	0.018	0.041	0.412	0.017	9962
**Chandigarh**	0.105	0.448	0.047	0.149	0.403	0.06	0.037	0.378	0.014	0.03	0.433	0.013	0.053	0.438	0.023	2640
**Dadra and Nagar Haveli**	0.122	0.402	0.049	0.382	0.445	0.17	0.158	0.437	0.069	0.057	0.386	0.022	0.273	0.442	0.121	3285
**Daman & Diu**	0.101	0.436	0.044	0.09	0.478	0.043	0.049	0.408	0.02	0.053	0.491	0.026	0.061	0.452	0.027	5612
**Delhi**	0.051	0.431	0.022	*- NA -*	*- NA -*	*- NA -*	0.054	0.407	0.022	0.035	0.486	0.017	0.044	0.441	0.019	21056
**Lakshadweep**	*- NA -*	*- NA -*	*- NA -*	0.008	0.375	0.003	*- NA -*	*- NA -*	*- NA -*	*- NA -*	*- NA -*	*- NA -*	0.008	0.395	0.003	3956
**Puducherry**	0.031	0.387	0.012	0.023	0.348	0.008	0.016	0.375	0.006	0.011	0.455	0.005	0.02	0.373	0.007	13380
**INDIA**	**0.292**	**0.473**	**0.138**	**0.444**	**0.486**	**0.216**	**0.245**	**0.465**	**0.114**	**0.149**	**0.463**	**0.069**	**0.248**	**0.471**	**0.117**	**2703773**

Note:—*NA -*: Data ‘*Not Available*’.

Source: Authors’ own estimation.

From Table 4 it can also be noticed that among the SCs, Bihar recorded the highest MPI, followed by Jharkhand, Uttar Pradesh, Madhya Pradesh, Rajasthan, Nagaland and Odisha. On the other hand, MPI is lowest in Kerala, Goa, Puducherry, Andaman and Nicobar Islands, Sikkim, Mizoram and Delhi. The highest MPI for the STs was recorded in Madhya Pradesh, Bihar and Uttar Pradesh; these states show almost similar MPI. Whereas lowest MPI was recorded in Puducherry followed by Lakshadweep, Sikkim, Andaman, Kerala and Goa. Data is not available for STs in Delhi and Punjab. Similarly, data on OBCs in Lakshadweep is not available. Among the OBCs across the states and UTs, the lowest MPI was recorded in Kerala followed by Goa and Puduchchery, meanwhile highest in Bihar followed by Uttar Pradesh and Jharkhand in OBC communities. The Others social group’s data were not available for Lakshadweep and Kerala. The states of Punjab, Puducherry and Tamil Nadu recorded the lowest MPI among the Others across the states and UTs, followed by Chandigarh, Goa and Sikkim, those have almost equal MPIs. The highest MPI for the Others social group is merely equal in Meghalaya, Bihar, Assam, Nagaland and Arunachal Pradesh, with Meghalaya slightly worse off than the above-mentioned states.

While comparing the ranks of 36 states and union territories in terms of Adjusted Headcount Ratio (M_0_) and Headcount Ratio (H) for SCs, it is observed that Arunachal Pradesh, Kerala, Meghalaya, and Nagaland were ranked higher in MPI than their ranks in H. This indicates a greater intensity of poverty (A) among the SCs in these states compared to states with H values closer to the respective states. Similarly, among the STs in Haryana, OBCs in Mizoram and Punjab, and Others in Daman & Diu marked a higher intensity of poverty compared to the states and UTs with a nearby value to their H.

At the national level, STs is the most deprived social group, followed by SCs according to the MPI estimates according to the MPI estimates, and half of the States and UTs (18 out of 36) follow the same pattern. On the other hand, in six states and UTs, the SCs are the most deprived, followed by STs: Bihar, Himachal Pradesh, Sikkim, Damn and Diu, and Puducherry. Contrarily, in Arunachal Pradesh, Assam, Meghalaya, Mizoram, and Andaman and Nicobar Island the Others social group were estimated to have greater values for adjusted headcount ratio (M_0_). In Uttarakhand and Delhi, both OBCs and SCs have the greater MPI values. States, where the STs and SCs outperform OBC and Others social groups in terms of MPI, are Arunachal Pradesh, Mizoram, and Andaman and Nicobar Island. It is also interesting to note that while STs of four of the eight north-eastern states (Arunachal Pradesh, Assam, Mizoram, and Nagaland) perform quite better compared to their other social group counterparts, in the rest four (Manipur, Meghalaya, Sikkim, and Tripura) STs’ MPI is noticeably greater than other social groups.

### 4.3. Decomposition of MPI by social groups, dimensions, and indicators

**Fig 2:**
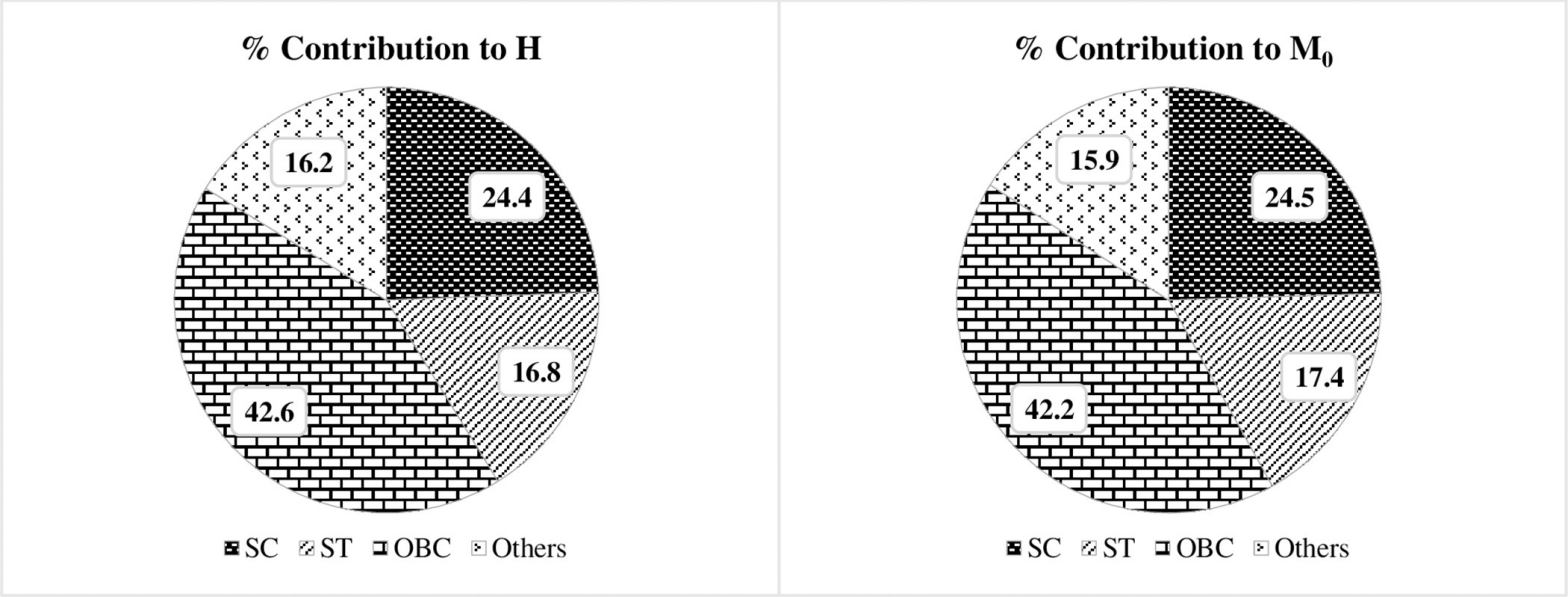
Social group-wise contribution to headcount ratio (H) and adjusted headcount ratio (M_0_).

**Social groups.** The property of dimensional breakdown is relevant to multidimensional poverty which allows poverty to be broken down by deprivations in all subgroups, dimensions, and indicators among the poor [41]. Fig 2 demonstrates the social group-wise contribution to H and M_0_ of India. It shows OBCs contribute more than two-fifths and SCs more than one-fifth to both H and M_0_, while the Others social group the least. However, when the population share of social groups is compared with their contribution, it is observed that STs contribute nearly twice their population share for both H and M_0_. And the SCs contribution is also noticeably higher than their population share. Whereas the contributions of OBCs and Others social groups is smaller than their population shares.

### Dimensions

**Fig 3:**
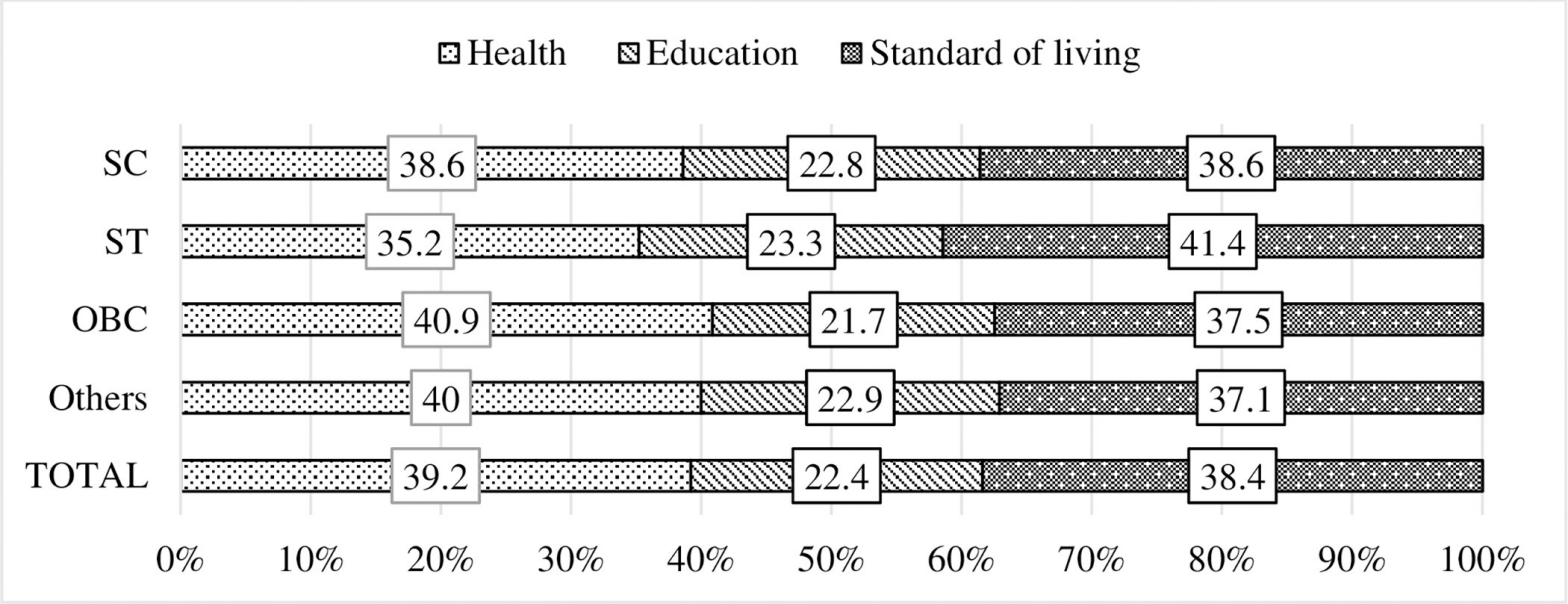
Decomposition of multidimensional poverty index by the three dimensions and by the social groups.

Fig 3 illustrates the contribution of each dimension to MPI across the castes. At the national level both standard of living (38.4%) and health (39.2%) are the major contributor to multidimensional poverty with almost equal contribution. The dimensional contribution varied noticeably across the social groups. While for STs, standard of living is the greatest contributor, followed by health, for OBCs and Others social groups health dimension is the most significant contributor, followed by standard of living. On contrary, both standard of living and health contributes equally to the MPI for SCs.

### Indicators

Table 5 depicts the contribution of each indicator to the MPI of each social group in India. Of the 12 indicators considered, the largest contribution to multidimensional poverty came from nutrition (28.4%), followed by years of schooling (14.9%), maternal health (9.7%), and cooking fuel (9.5%). And except for STs, all social groups show a similar order of contribution to MPI. For the STs, the maternal health’s contribution to the MPI is preceded by cooking fuel, sanitation and housing. It is also noticed, while drinking water contributes merely 2% to the MPIs of SCs, OBCs and Others, it contributes relatively higher proportion (4%) to the MPI for STs. While all of the 12 indicators show a similar pattern of contribution to MPIs of SCs, OBCs and Others, the contribution of nutrition and maternal health indicator to MPI of STs is noticeably lower, and the contribution of safe drinking water and household assets is remarkably higher compared to rest of the social groups.

**Table 5:** Contribution of each indicator to multidimensional poverty index across social groups.

Dimension	Indicators	SC	ST	OBC	Others	Total
**Health**	Nutrition	28	26.4	29.3	29	28.4
	Child & Adolescent mortality	1.1	0.8	1.2	1.1	1.1
	Maternal Health	9.5	8	10.4	10	9.7
	**Overall**	**38.6**	**35.2**	**40.9**	**40.1**	**39.2**
**Education**	Years of Schooling	15.3	15.6;	14.3	14.9	14.9
	School Attendance	7.5	7.7	7.3	8	7.5
	**Overall**	**22.8**	**23.3**	**21.6**	**22.9**	**22.4**
**Standard of living**	Cooking Fuel	9.5	9.6	9.5	9.3	9.5
	Sanitation	9	9	8.8	8.1	8.8
	Drinking Water	1.9	4	1.9	2	2.3
	Electricity	3.7	3	3.7	3	3.5
	Housing	8.6	8.8	8.4	8.3	8.5
	Assets	3.7	4.9	3.2	3.7	3.7
	Bank Account	2.2	2.2	2	2.8	2.2
	**Overall**	**28.6**	**41.5**	**37.5**	**37.2**	**38.5**

**Note:** The overall values may not be summed to get 100%, as the values were rounded to their nearest integers.

**Source:** Authors’ own estimation.

## 5. Discussion

The study’s objective is to assess India’s multidimensional poverty across social groups. This analysis adds to existing base of information in India about household-level multidimensional poverty measurement. This study contributed to a better understanding of poverty in India by offering a detailed analysis and data disaggregation in line with the SDGs. To the best of our knowledge this is the first ever study that comprehensively estimates multidimensional poverty among social groups across India and its states and UTs, and examines the contribution of indicators and dimensions to MPI in India as well as to MPI of the social groups. The salient findings of the study are discussed in this section.

The results reveal that nearly one-fourths of Indian population are living in households that considered as multidimensionally poor. The extent and nature of multidimensional poverty vary significantly across the four social groups in the population as well as across the States and UTs. The highest MPI was estimated for states located in the central region: Bihar, Jharkhand, Uttar Pradesh, Madhya Pradesh and Chhattisgarh; While Assam, Meghalaya, Rajasthan, Odisha and Nagaland are among others with higher MPI. Tripathi & Yenneti [42] have also observed these states have the greater MPIs but with different orders. and this finding is in line with Dehury & Mohanty [31]. These 10 states account for nearly three-fifths (57.1%) of the Indian population [43], hence have the potential to alter the multidimensional poverty at the national level. In addition, the states in the central region recorded the highest levels of multidimensional poverty among all social groups. For these reasons, this study suggests that there is a need for in-depth assessment of poverty in these states to uncover poverty conditions, improve the effectiveness of evidence-based planning and for effective policy making. In this way, interventions can be adapted to account for the heterogeneities of states and regions, as well as improve targeting of policy interventions. The lowest MPI were estimated to be in the state of Kerala, this finding is supported by Dehury & Mohanty [31] and Tripathi & Yenneti [42]. Other than Kerala, Puducherry, Lakshadweep, Sikkim, Goa, Delhi and Tamil Nadu have remarkably lower MPI compared to other states and UTs.

Regarding the social groups differential in MPI, this study found ST as the most deprived social group, with a dramatically higher number of multidimensionally poor people compared to their other social group counterparts, and Others social group are the most prosperous. This finding is consistent with the findings of the studies conducted by Bagli & Tewari [44] in Purulia district of West Bengal, Alkire et al. [33] in India, and in a recent study in urban India by Kaibarta et al. [45]. Our result that SCs and OBCs have nearly equal rates of multidimensional poverty is disproved by findings from another study [46], which show that OBCs have a far lower rate than SCs. When it comes to decomposing MPI across social groups, OBCs contribute the most, which can be linked to their population share being the highest of all social groups. However, it is worth noting that the STs contribute a significantly higher amount of the national MPI, around double their population share, whilst the SCs contribute around 1.25 times their population share. OBC and Other contributions, on the other hand, are lower than their population proportions.

MPI decomposition by dimensions and indicators yields some intriguing results. Both standard of living and health are key contributors to multidimensional poverty at the national level, with almost equal contributions. In their studies of 82 natural regions in India and urban India, Dehury & Mohanty [31] and Mohanty & Vasishtha [46], respectively, revealed that the health dimension provides the largest share to MPI, which is consistent with our findings. In contrast to our findings, Mohanty and Vasishtha [46] claim that standard of living has the smallest impact on MPI. The dimensional contribution varied noticeably between social groups. For OBCs and Others social groups health dimension contributes the highest, followed by standard of living. Whereas, for STs, standard of living is the greatest contributor, followed by health, which supported by the findings of Kaibarta et al. [45]. This finding backs up by Megbowon [46] and Espinoza-Delgado & Klasen [18], who argued that the standard of living dimension contributes the most to multidimensional poverty, particularly in poorer countries and rural areas. Because STs in India are usually located in hard-to-reach places and have historically been economically poorer than others, which restricts them from availing improved facilities in the standard of living dimension. On the contrary, for SCs, both standard of living and health play an equally role in MPI. Though SCs, like STs, are mostly located in rural areas and economically weaker, they have been settled closer to the society’s mainstream, allowing them to exercise their basic rights and adapt to better amenities to a larger extent than STs.

The decomposition of MPI with respect to the 12 indicators revealed that, the largest contribution to multidimensional poverty came from nutrition, followed by years of schooling, maternal health, and cooking fuel. Except for STs, all social groups, follow the similar sequence of contribution by 12 indicators to the MPI at the national level. While nutrition and years of schooling are greater concerns for STs’ MPI, unlike other three social groups the contribution of maternal health is preceded by cooking fuel, sanitation, and housing. In overall, the study recommends, the government should allocate more resources to improve the nutritional and health status of its citizens as well as their educational level and attainment. Because education is a critical area and its absence and inadequacy prevent individuals as well as the concerned households from realizing their basic rights [40]. Similarly, health has both intrinsic and instrumental value as well [22]; because it can affect several others capabilities, for instance, being not healthy can limit an individual’s capability to take part in social activities and prevent them to practice their profession [47].

## 6. Conclusion

In this era of globalization and the information technology revolution, poverty has no longer been considered as an absolute concept measured in money metrics. It has been realized that people may have better income, but may be lacking in health, nutrition or may not access proper day to day facilities or education or in any other dimension which prevents them to benefit from the mainstream economy. So, we call them multidimensional poor. This study explored the multidimensional poverty problem among social groups across India. The significant factors contributing to it are household used unimproved fuel for cooking purposes, unimproved sanitation facilities, lack of proper nutritional intake of children or men or women, improper maternal health and lack of education on the household level. The analysis across several categories provides essential information to policymakers since it indicates the particular areas of who the poor are, where they live, and how poor they are. Based on this finding, the policy should be formulated according to the deprivation pattern identified among the four social groups in each state with specifically designed plans and programs. Provision of providing access to clean cooking fuels, affordable and improved sanitation, providing safe and accessible drinking water, better free healthcare services, promote education for all, aid housing assets accumulation for the poor, electricity subsidies coverage etc. would facilitate in reducing the headcounts as well as the intensity of multidimensional poverty in these respective areas so that its economy may be placed on a high growth trajectory. Furthermore, disaggregating data analysis by these different segments allows for monitoring of the SDG’s commitment to halve the number of men, women, and children living in poverty in all of its aspects, as well as the ‘Leaving No One Behind’ commitment. From a multidimensional deprivation standpoint, the findings of this social group-based poverty study are not only an improvement over previous poverty measures that use money, but they also serve to address a knowledge gap. As emphasised by SDGs target 1.3, the Alkire-Foster methodology [23] for constructing the household-level multidimensional poverty measure assists policymakers in examining the poor’s joint deprivation, allowing them to implement nationally appropriate social protection systems and cover those left behind.
